# Time-to-event versus ten-year-absolute-risk in cardiovascular risk prevention – does it make a difference? Results from the Optimizing-Risk-Communication (OptRisk) randomized-controlled trial

**DOI:** 10.1186/s12911-016-0393-1

**Published:** 2016-11-29

**Authors:** Charles Christian Adarkwah, Nikita Jegan, Monika Heinzel-Gutenbrunner, Felicitas Kühne, Uwe Siebert, Uwe Popert, Norbert Donner-Banzhoff, Sarah Kürwitz

**Affiliations:** 1 Department of General Practice and Family Medicine, Philipps-University, Marburg, Germany; 2 CAPHRI School for Public Health and Primary Care, Department of Health Services Research, Maastricht University, Maastricht, The Netherlands; 3 Department of Child and Adolescent Psychiatry, Philipps-University, Marburg, Germany; 4 Institute of Public Health, Medical Decision Making and Health Technology Assessment, Department of Public Health and Health Technology Assessment, UMIT - University for Health Sciences, Medical Informatics and Technology, Hall i.T., Austria; 5 Division of Public Health Decision Modelling, Health Technology Assessment and Health Economics, ONCOTYROL - Center for Personalized Cancer Medicine, Innsbruck, Austria; 6 Center for Health Decision Science, Department of Health Policy and Management, Harvard School of Public Health, Boston, MA USA; 7 Department of General Practice, Georg-August-University, Göttingen, Germany; 8 Department of Public Health, University of Bielefeld, Bielefeld, Germany

**Keywords:** Randomized-controlled trial, Arriba^TM^, Decision-aid, Shared decision-making, Ten-year-prognosis, Risk-assessment, Lifetime risk, Time-to-event, Cardiovascular disease, Decisional conflict, Accessibility

## Abstract

**Background:**

The concept of shared-decision-making is a well-established approach to increase the participation of patients in medical decisions. Using lifetime risk or time-to-event (TTE) formats has been increasingly suggested as they might have advantages, e.g. in younger patients, to better show consequences of unhealthy behaviour. In this study, the most-popular ten-year risk illustration in the decision-aid-software arriba^TM^ (emoticons), is compared within a randomised trial to a new-developed TTE illustration, which is based on a Markov model.

**Methods:**

Thirty-two General Practitioners (GPs) took part in the study. A total of 304 patients were recruited and counseled by their GPs with arriba^TM^, and randomized to either the emoticons or the TTE illustration, followed by a patient questionnaire to figure out the degree of shared-decision-making (PEF-FB9, German questionnaire to measure the participation in the shared decision-making process, primary outcome), as well as the decisional conflict, perceived risk, accessibility and the degree of information, which are all secondary outcomes.

**Results:**

Regarding our primary outcome PEF-FB9 the new TTE illustration is not inferior compared to the well-established emoticons taking the whole study population into account. Furthermore, the non-inferiority of the innovative TTE could be confirmed for all secondary outcome variables. The explorative analysis indicates even advantages in younger patients (below 46 years of age).

**Conclusion:**

The TTE format seems to be as useful as the well-established emoticons. For certain patient populations, especially younger patients, the TTE may be even superior to demonstrate a cardiovascular risk at early stages. Our results suggest that time-to-event illustrations should be considered for current decision support tools covering cardiovascular prevention.

**Trial registration:**

The study was registered at the German Clinical Trials Register and at the WHO International Clinical Trials Register Platform (ICTRP, ID DRKS00004933); registered 2 February 2016 (retrospectively registered).

**Electronic supplementary material:**

The online version of this article (doi:10.1186/s12911-016-0393-1) contains supplementary material, which is available to authorized users.

## Background

Shared-decision-making (SDM) of patients and health professionals is increasingly becoming the norm for health related decisions. SDM was formulated in the mid-nineties by a Canadian working-group [1]. It is defined as a process of interaction between patient and physician, both equally and actively participating, well-informed, with the goal to achieve a common agreement based on the information available [2]. Studies show that an increased participation in the decision-making process leads to a greater satisfaction of doctors and patients and to a better adherence and clinical outcomes [3, 4].

Decision-aids are designed to help patients make informed choices by delivering evidence-based information on options and outcomes, e.g. regarding cardiovascular events [5]. They typically cover diagnostic, therapeutic and preventive decisions and are also able to inform the counselling process as they can be delivered in different formats before, during or after the consultation [6]. Decision-aids are reported to increase knowledge, reduce decisional conflict, cause greater satisfaction with decision-making, support more realistic expectations, achieve a greater likelihood of being able to make a decision, result in an increased association between patient values and decisions, support patient participation, and enhance communication between physicians, patients and their relatives [7, 8].

Cardiovascular diseases are a burden for all healthcare systems, especially in the Western world. Thus, predicting cardiovascular risk and tailoring preventive efforts accordingly is recommended by national guidelines [9, 10].

Prediction tools are used for this purpose to demonstrate risk and might initiate behavioural change. They usually provide individualized absolute risk estimates for a limited time period, such as 10 years. This format has been criticized, however, for underestimating high-risk constellations in the young and middle aged [11–13]. These age groups may have unfavourable risk profiles relative to their age group. However, since their absolute risk is still low, this does not become obvious and an opportunity for early intervention may be missed [11]. To overcome this problem, lifetime-risk and time-to-event formats have been suggested [9, 11–15].

In order to systematically compare both formats, we developed two risk-displays as part of the computerized decision-aid arriba^TM^. The decision-aid-software is well-established in Germany [16] and being introduced also in other European countries. It has been extensively tested and found to be a valuable tool in primary care practice [17–20].

Within arriba^TM^, the calculation of absolute cardiovascular risks is based on the Framingham risk algorithm [21, 22]. The TTE-displays evaluated in this study based on a Markov-model [23], which was constructed for the purpose of our study (unpublished data). A Markov model is an iterative process where patients are assumed to stay in one cycle (i.e., a defined health state) for a certain time and then make a transition to another cycle. Markov models are useful when a decision problem involves risk that is continuous over time, when the timing of events is important, and when important events may happen more than once. Model parameters and justification are available upon request.

The 10-year-absolute risk format can be visualized with emoticons (Fig. 2). For the time to event prediction, we developed a display based on a time bar combined with a point estimated for possible events (Fig. 3). These displays emerged as the most accessible and accepted from a series of exploratory studies comparing different presentation formats for cardiovascular risk. (unpublished data).

Following the guidelines for complex interventions [24–26], we aim to compare the new TTE illustration with the established emoticons looking at the degree of SDM in the consultation process and various secondary outcomes, amongst others the decisional conflict and accessibility. Additionally, we closely look at age-dependent differences to figure out if the hypothesis mentioned above regarding a potential advantage of a lifetime risk illustration in younger patients.

## Methods

### Design and setting

This prospective, cluster-randomized trial was performed in general practices in the greater area of Marburg, Germany between October 2012 and January 2013. The study was performed in accordance with the Declaration of Helsinki and approved by the research ethics committee of the University of Marburg. The study was registered at the German Clinical Trials Register (DRKS-ID: DRKS00004933).

### GP recruitment

**Fig. 1:**
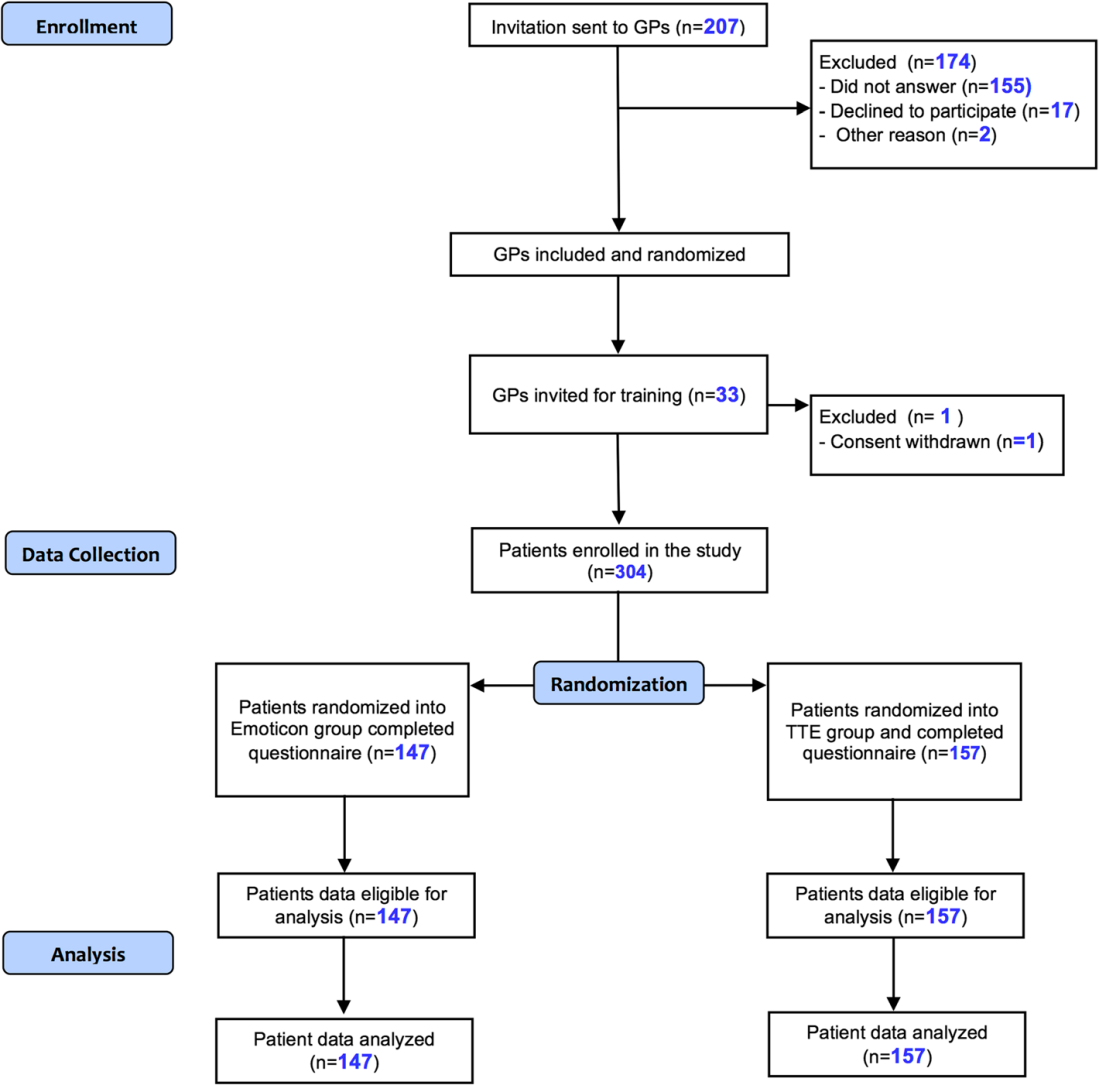
Flowchart of participation

A convenience sample of GPs who are affiliated with the Department of Primary Care at the University of Marburg were invited by mail to take part in the study (see Fig. 1 for recruitment details). Thirty-two GPs in twenty-eight practices agreed to participate in the study. They took part in meetings to be trained in study procedures and the use of both ways of showing risk in arriba^TM^, the emoticons and the TTE, Each GP received advice on how to communicate risk according to the respective risk format and how to make use of the material in arriba^TM^. Immediately after the training they completed a questionnaire regarding personal characteristics and workload as well as prior experience with the decision-aid arriba^TM^. Finally, they received a booklet summarizing the content of the training for further reading and individual preparation. The training was followed by an office visit of a study nurse in order to install the study software, handover the study material and clarify any issues with the GPs. In addition, GPs could contact the study group anytime in case of problems or doubts about any issue during the study period. The GPs received a financial compensation for every patient included in the study.

### Patient recruitment

Patients were eligible for the study if they were aged 30 to 80 years and the GP felt a need to discuss behavioral change regarding cardiovascular risk. This could be, for example, the biannual health check “CheckUp 35+”, offered to adults above 35 years, a disease management program (DMP) consultation or a discussion of medication after specialist consultation. Patients were also eligible, if they addressed cardiovascular risk and possible prevention themselves. The latter could include medications (statins, low-dose aspirin, antihypertensive drugs or dose adjustments), dietary changes, exercise or smoking. Patients were excluded if they were – according to the judgement of the GP – significantly impaired cognitively, had insufficient knowledge of the German language or had no interest in taking an active part in the decision process.

### Interventions

**Fig. 2:**
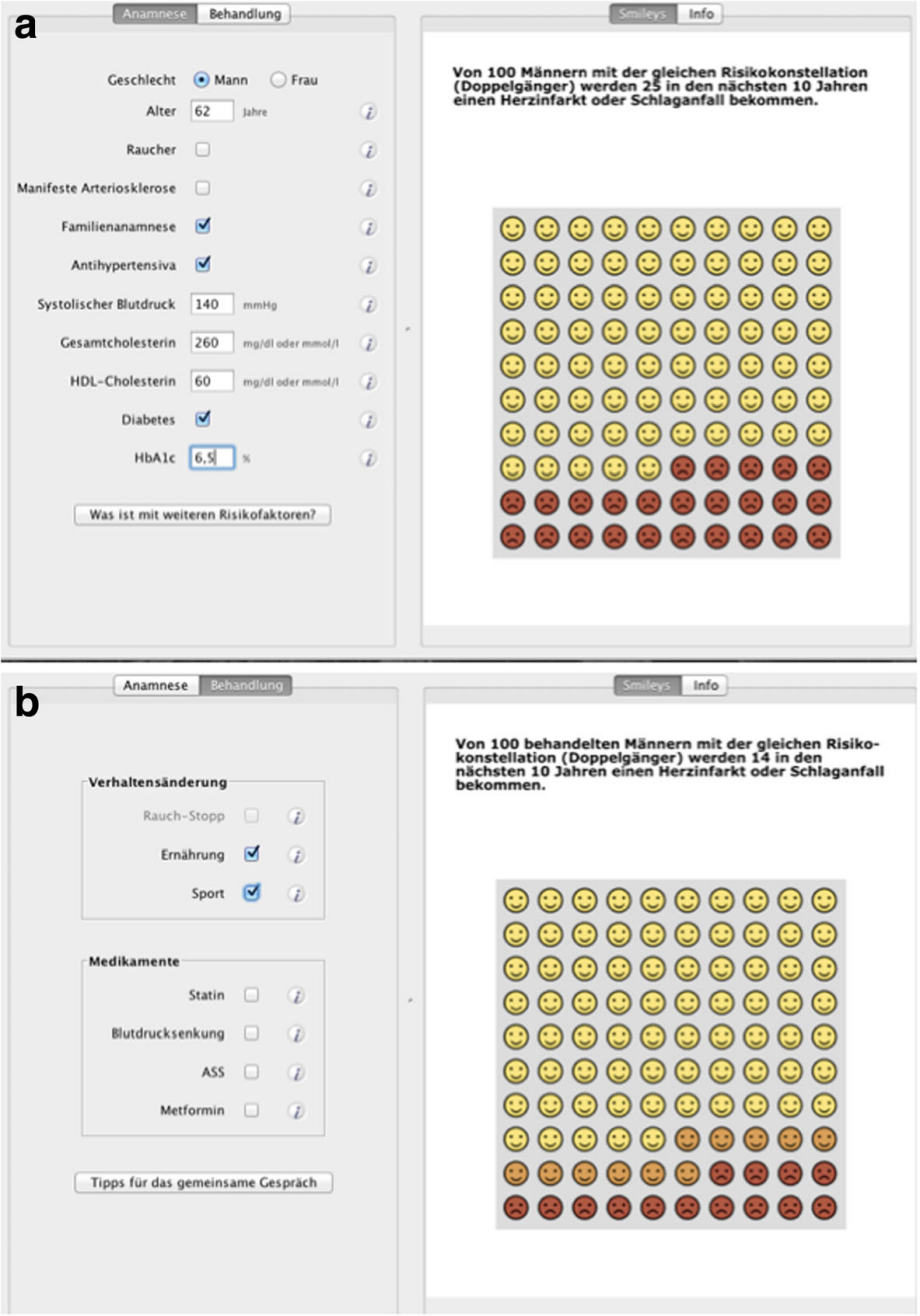
Emoticon-interface of the arriba^TM^ decision-aid (**a** Medical history taking^1^, **b** visualization of treatment options^2^) Footnote: ^1^On the left, information regarding the medical history must be filled in, which is (from top to bottom): gender, age, smoking-status, presence of manifest arteriosclerosis, positive family history, taking of antihypertensive medications, systolic blood pressure, total cholesterol level, high density lipoproteins (HDL) level, presence of diabetes, HbA1c level. On the right, the emoticons are displayed, accompanied by the following headline: “Out of 100 men with the same risk profile, 25 will suffer a myocardial infarction or stroke within the next 10 years.” ^2^On the left, the treatment options are shown, which are (from top to bottom): behavioral changes, like smoking cessation, nutrition, sports and on the other hand drug treatment, i.e. statins, antihypertensive drugs, aspirin, metformin. On the right, the emoticons are displayed, accompanied by the following headline: “Out of 100 treated men with the same risk profile, 14 will suffer a myocardial infarction or stroke within the next 10 years.” The risk reduction (here due to nutrition modification and sports) is indicated with orange-colored emoticons

**Fig. 3:**
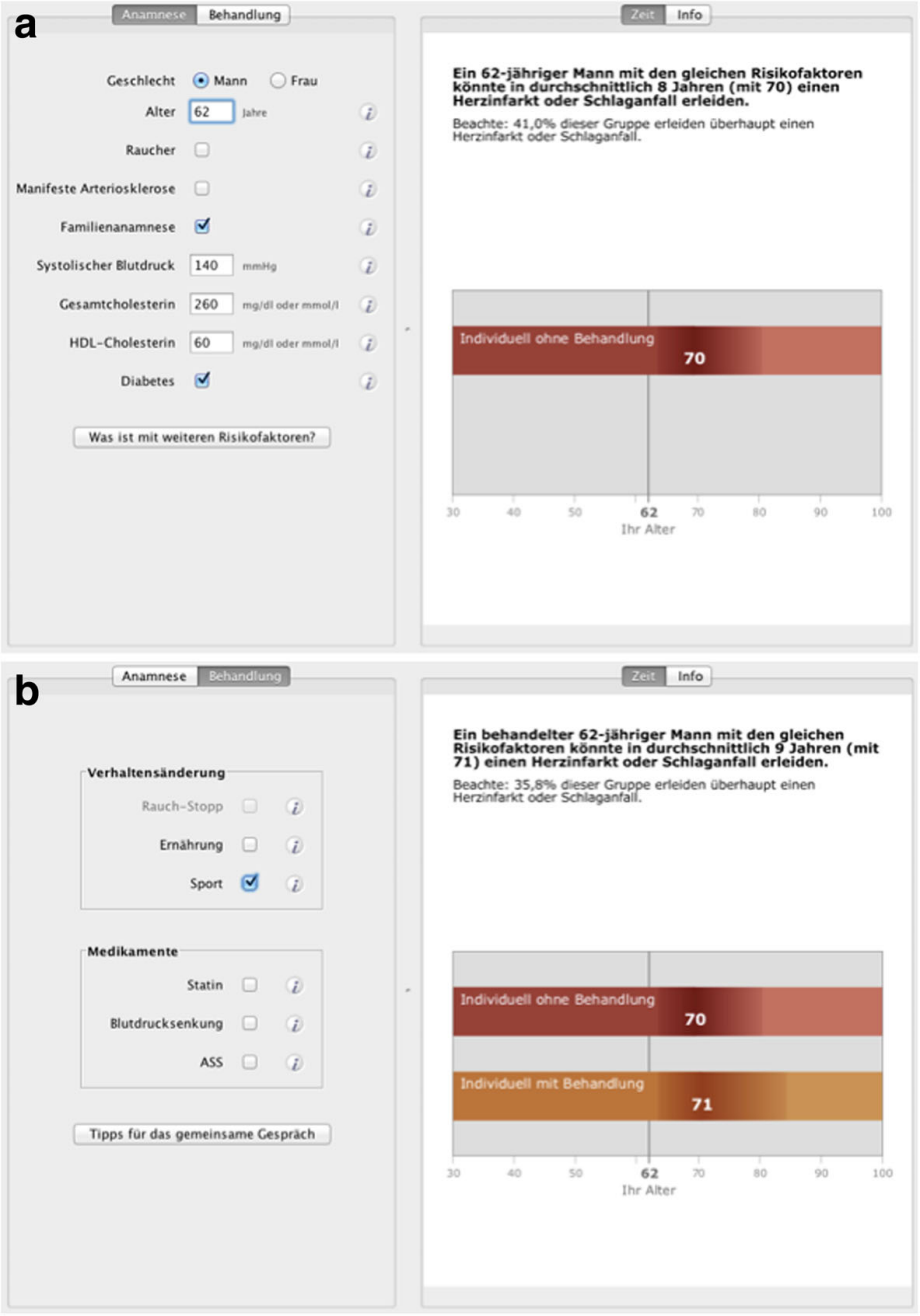
TTE-interface of the arriba^TM^ decision-aid (**a** Medical history taking^3^, **b** visualization of treatment options^4^) Footnote: ^3^On the left, the information regarding the medical history must be filled in, which is (from top to bottom): gender, age, smoking-status, presence of manifest arteriosclerosis, positive family history, taking of antihypertensive medications, systolic blood pressure, total cholesterol level, high density lipoproteins (HDL) level, presence of diabetes. On the right, the TTE graph is displayed, accompanied by the following headline: “A 62y old man with the same risk profile could suffer a myocardial infarction or stroke in in average time of 8 years form now (at 70y of age). ^4^On the left, the treatment options are shown, which are (from top to bottom): behavioral changes, like smoking cessation, nutrition, sports and on the other hand drug treatment, i.e. statins, antihypertensive drugs, aspirin. On the right, TTE graphs are displayed, accompanied by the following headline: “A 62y old treated man with the same risk profile could suffer a myocardial infarction or stroke in in average time of 9 years form now (at 71y of age). The risk reduction (here due to sports) is indicated with the orange-colored timeline. The following statistical information is added: “35,8% of this group suffer a myocardial infarction or stroke at all”

Immediately after giving their informed consent, patients were randomized to consultation with the emoticons (Fig. 2) or the TTE illustration (Fig. 3). GPs entered a study ID into the decision support software, which automatically allocated each patient into one of the two conditions according to an a priori randomised sequence. GPs learned about each patient’s allocation by the illustration displayed by the software. They then started a discussion with their patients on the basis of the allocated display, i.e. either emoticons, or TTE, respectively. After the consultation, patients were asked to fill in a questionnaire covering the immediate outcome assessments. GPs recorded the decision made, such as specific medications, dose adjustments, behavioral measures or no change at all. Three months later, patients were contacted by telephone to assess their adherence. The results of these follow-up interviews will be published elsewhere.

### Outcomes

#### Primary outcome

The main objective of the study was to evaluate the non-inferiority of the new TTE illustration compared to the emoticons regarding their impact on SDM. This was operationalized by the German questionnaire on shared-decision-making (SDM), the PEF-FB-9 (PEF is the German acronym for SDM). This instrument [27] measures the degree of participation which patients experience in the consultation as seen by the patient him- or herself. The instrument has been evaluated for its psychometric characteristics [27]. Moreover, it has been applied in various studies on different patient groups and clinical topics [28–30].

#### Secondary outcomes

In addition to the primary endpoint mentioned above, we evaluated several secondary endpoints. First, the decisional conflict was assessed by the German version of the Decisional Conflict Scale (DCS) [31, 32]. The DCS encompasses sixteen items, measured on a five-point Likert scale. The decisional conflict scale measures personal perceptions of uncertainty in choosing options, modifiable factors contributing to uncertainty such as feeling uninformed, unclear about personal values and unsupported in decision making and finally effective decision making such as feeling the choice is informed, values-based, likely to be implemented and expressing satisfaction with the choice [33].

Second, the degree of information was measured, using the German version of the Preparation for Decision-Making Scale (PDMS-D) [34]. The PDMS-D contains ten items and includes two dimensions: Seven items are related to the preparation for the decision-making itself and three items capture the patient’s preparation for the GP consultation. According to the authors of the PDMS Preparation for Decision Making is defined as ‘a patients’ perception of how useful a decision aid or other decision support intervention is in preparing the respondent to communicate with their practitioner at a consultation visit and making a health decision“ [35].

While the PDMS-D focuses more on the information material used in the counseling process, the DCS accounts for the quality of the decision.

Third, subjective accessibility of the information was assessed using an adaptation of the scale by Gaissmeier and colleagues [36]. This encompasses four questions at a five-point Likert scale, ranging from 1 (*not at all*) to 5 (*very much*), asking about the four aspects of information: comprehensibility, usefulness, seriousness and intuitive accessibility. Answers were averaged to generate one accessibility score for each participant.

Finally, patients were shown two visual analog scales in order to rate on the one hand the importance of avoiding a cardiovascular event and on the other hand to estimate their own cardiovascular risk.

### Statistical analyses

All statistical calculations were performed with IBM SPSS [37]. We calculated means, standard deviations and frequencies for the descriptive data. The hypotheses concerning the non-inferiority of the TTE illustration are tested by means of confidence intervals based non-inferiority tests according to the following equation:

$$ \mathrm{H}1:\upmu 1\hbox{-} \upmu 2 < \upvarepsilon $$

with μ1 as the score mean or the total score of the particular question in the emoticon group and μ2 accordingly in the TTE group, while ε is the pre-specified non-inferiority margin. We defined the boundary ε, as 1/10 of the range of the respective questionnaire-score. The non-inferiority tests are performed by calculating a one-sided 95%-confidence interval for the difference between μ1 and μ2. If the upper bound of this confidence interval is less than ε, it can be concluded that the TTE illustration is not inferior compared to the emoticons. In order to examine the importance of the differences between the two graphic dentitions, effect sizes (Cohen’s D) are calculated [38].

We compare the two illustrations in respect to one primary outcome variable, PEF-FB9, and additionally to several secondary outcome variables (amongst others the decisional-conflict, preparation for decision-making, risk perception). An additional secondary analysis is the exploration of a potential superiority of the new TTE illustration in younger patients, less than 46 years of age. This is investigated by testing the interaction term of age-group and illustration in a two-factorial analysis of variance, with the factors “age-group” and “illustration”. For this analysis the population is split up into two age-groups, <=45 years and > 45 years. Finally, for the patients younger than 46 years, the average of all questions or test-scores will be compared descriptively regarding the two illustrations.

In line with the exploratory nature of the tests concerning the secondary outcome variables, we apply no alpha-correction for multiple testing. If the confidence-interval-based test happens to suggest superiority of the new TTE illustration, it is justifiable to state the result of superiority [39].

Characteristics of the patients are demonstrated by mean, standard errors in metric variables, frequencies and percentages in categorical variables. Non-inferiority hypotheses are performed by means of one-sided 95% confidence intervals. The alternative hypothesis of non-inferiority is accepted, if the lower bound of the confidence interval for the difference of μ_1_ μ is < ε. Superiority of the TTE in respect of the decisional conflict in young patients is explored by means of a two-way ANOVA. A significant interaction term of age-group and presentation would indicate superiority of the TTE illustrations over the emoticons.

## Results

Thirty two GPs in 28 practices included an average of 9.5 patients (range from 3 to 15) which resulted in a sample of 304 study participants (Fig. 1). 147 patients were shown emoticons and 157 patients received their risk information on the basis of the TTE illustration. The characteristics of the two groups (emoticons and TTE) are shown in Table 1. This is a sample of predominantly older, male patients with known risk factors and comorbidities. Apparently this is the group, where GPs and/or patients feel a need to discuss cardiovascular prevention, both study arms were well-balanced regarding sociodemographic and clinical variables.

**Table 1:** Characteristics of the study population

	Emoticons	Time-to-event (TTE)
Age (yr), M (SD)	58.01 (10.66)	57.83 (11.033)
Proportion of immigrants, n (%)	11 (8)	14 (9)
Proportion of pts with low level of education^1^	66 (45%)	67 (43%)
Gender: male, n (%)	88 (60%)	87 (55%)
Known hypertension, n (%)	36 (25%)	45 (29%)
Total cholesterol level > 200 mg/dl n (%)	99 (67%)	115 (73%)
HDL < 40 mg/dl, n (%)	25 (17%)	21 (13%)
Known Diabetes, n (%)	43 (29%)	41 (26%)
Current smoker, n (%)	40 (27%)	43 (27%)
Male age >55, female age >65, n (%)	67 (46%)	70 (45%)
Known vascular disease^2^, n (%)	15 (10%)	14 (9%)
Family history of vascular disease^3^, n (%)	49 (33%)	54 (34%)
Hypertension medication, n (%)	84 (57%)	76 (48)
Mean number of risk factors	3.08 (1.57)	3.01 (1.33)
Patients with arriba experience, n (%)	15 (10%)	17 (11%)

*Yr* year, *pts* patients, *HDL* high density lipoprotein cholesterol

^1^Low level of education definded as no general certificate of secondary education

^2^Evidence of either coronory heart disease, stroke or peripheral arterial occlusive disease

^3^At least one first-degree relative with coronary heart disease, occured before the age of 55 in men, and 65 in women

### Primary outcome

The mean difference of emoticons and TTE regarding the PEF-FB9 is −1.69 with a 90%-confidence interval ranging from −5.51 to 2.12. Thus, the upper limit of the 90%-confidence interval is clearly below the pre-specified non-inferiority margin ε, which is 10 (10% of the scale’s total range of 100). In addition, the effect size according to Cohen is d = 0.09, which represents a small effect (see Table 2). Taken together, these two results confirm the non-inferiority of the TTE-display. Regarding our primary outcome PEF-FB9 the TTE illustration is not inferior compared to the emoticons. There is no significant difference to be found between the two ways of presenting risk, taking the whole study population into account.

**Table 2:** Results of the non-inferiority tests

a. For all variables for which high values are favorable, the upper limit of one-sided 95% CI is relevant)							
Test-score	Mean (std) Emoticons	Mean (std) TTE	Max. Score	Non-inferiority margin ε	Upper limit of one-sided 95% CI	Non-inferiority confirmed	Effect size (Cohen’s d)
PEF-FB 9 Total score (primary outcome)	81.88 (20.58)	83.57 (18.12)	100	10	2.12	yes	0.09
Risk perception*	4.06 (2.344)	5.22 (2.83)	10	1	−0.659	Yes, even superiority	0.45
Importance of avoiding a CV event	8.84 (2.39)	9.06 (1.95)	10	1	0.19	yes	0.10
Accessibility Total Score	18,10 (1,835)	17,78 (2,598)	16	1.6	0.74	yes	−0.14
Subscore “comprehensibility”	4.69 (0.52)	4.56 (0.719)	4	0.4	0.245	yes	−0.21
Subscore “usefulness”	4.57 (0.598)	4.43 (0.74)	4	0.4	0.268	yes	−0.21
Subscore “seriousness”	4.4 (0.669)	4.46 (0.75)	4	0.4	0.074	yes	0.08
Subscore “intuitive accessibility”	4,40 (0.79)	4,34 (0.78)	4	0.4	0.195	yes	−0.08
PDMS-D total score	72,32 (19.57)	73.57 (19.89)	40	4	2.6	yes	0.06
subscore preparation for decision-making	74.34 (19.08)	74.74 (19.3)	40	4	3.29	yes	0.02
subscore preparation for GP consultation	68.91 (24.0)	70.5 (25.0)	40	4	3.16	yes	0.06
b. For all variables for which high values are not favorable, the lower limit of one-sided 95% CI is relevant							
Test-score	Mean (std) Emoticons	Mean (std) TTE	Max. score	Limit of equivalence	lower limit of one-sided 95% CI	Non-inferiority confirmed	Effect size (Cohen’s d)
DCS Total score	15.38 (13.96)	14,77 (12.36)	100	−10	−2.0	yes	−0.05
Informed Subscore	16,84 (17.79)	16.23 (16.01)	100	−10	−2.64	yes	−0.04
Values Clarity Subscore	14.81 (16.71)	13.64 (13.96)	100	−10	−1.76	yes	−0.08
Support Subscore	16,19 (15.93)	17,43 (16.45)	100	−10	−4.4	yes	0.08
Uncertainty subscore	15,57 (17.02)	14,73 (15.69)	100	−10	−2.27	yes	−0.05
Effective decision subscore	13.66 (15.85)	13.74 (15.31)	100	−10	−3.07	yes	0.00

DCS: Decisional Conflict Scale, high score stands for high level of decisional conflict

*PEF-FB 9* Shared decision-making questionnaire, 9 items; high score stands for high level of patient involvement

*CV* cardiovascular, *PDMS-D* Preparation for Decision-Making Scale – German version, *GP* general practitioner

*Risk perception is significantly higher in the TTE illustration group compared to the emoticons (*p* < 0.005)

### Secondary outcomes

The results of all secondary outcomes are shown in Table 2. Both the confidence intervals as well as the small effect sizes show that the non-inferiority of the innovative TTE could be confirmed also for the remaining outcome variables. In addition, both illustrations were rated as highly accessible.

Looking at the risk perception, the subjective risk was rated significantly higher in the TTE group compared to the emoticons. Here, the upper limit of the 90% confidence interval for μ1-μ2 is even smaller than 0, which shows the superiority of the TTE with respect to the risk perception. For this item, the effect size is 0.45, which represents a medium effect.

### Interaction with age

**Fig. 4:**
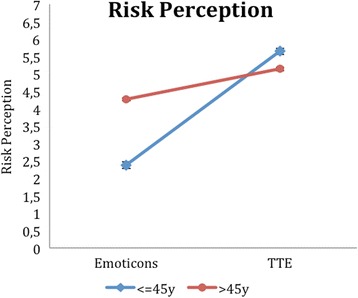
Risk perception as a function of age-group and illustration

As expected, the two age-groups vary in the numbers of risk factors, and their composition. Diabetes and getting antihypertensive treatment is more common in the elderly. Additionally, the percentage of smokers is higher in the elderly, while there are no significant differences in the proportion of male participants or migrants (see Table 3 for further details). The two illustrations have different effects depending on the patient’s age. We could see that providing information about the time-to-event instead of giving a 10-year absolute risk leads to a significant difference in the perceived risk. This was mainly the case in younger patients (<46 years) and shows that those patients counseled with the TTE experience a higher subjective risk to suffer a cardiovascular event (Additional file 1: Table S1, Fig. 4). This however does not impact the importance to avoid a cardiovascular event overall for which the interaction was not significant. For instance, this is most likely due to a ceiling effect.

**Table 3:** Characteristics of the study population, according to age-group

	pts < 45 years	pts > 45 years	*p*-value
Age (yr), M (SD)	39.79 (3,87)	60,58 (8,27)	<0,0005
Proportion of immigrants, n (%)	4 (10,5%)	21 (8%)	0,401
Proportion of pts with low level of education^1^	11 (28%)	122 (46%)	0.039
Gender: male, n (%)	21 (54%)	154 (58%)	0,608
Known hypertension, n (%)	7 (18%)	74 (28%)	0,245
Total cholesterol level > 200 mg/dl n (%)	23 (59%)	191 (72%)	0.131
HDL < 40 mg/dl, n (%)	8 (21%)	38 (14%)	0.338
Known Diabetes, n (%)	3 (8%)	81 (31%)	0.002
Current smoker, n (%)	16 (41%)	67 (25%)	0.053
Known vascular disease^2^, n (%)	4 (10%)	9 (9%)	0.775
Family history of vascular disease^3^, n (%)	16 (41%)	87 (33%)	0.365
Hypertension medication, n (%)	11 (28%)	149 (56%)	0.002
Mean number of risk factors	2.21 (1.32)	3.17 (1.43)	0.229
Patients with arriba experience, n (%)	1 (3%)	31 (12%)	0.096

*pts* patients, *yr* year, *pts* patients, *HDL* high density lipoprotein cholesterol

^1^Low level of education definded as no general certificate of secondary education

^2^Evidence of either coronory heart disease, stroke or peripheral arterial occlusive disease

^3^At least one first-degree relative with coronary heart disease, occured before the age of 55 in men, and 65 in women

**Fig. 5:**
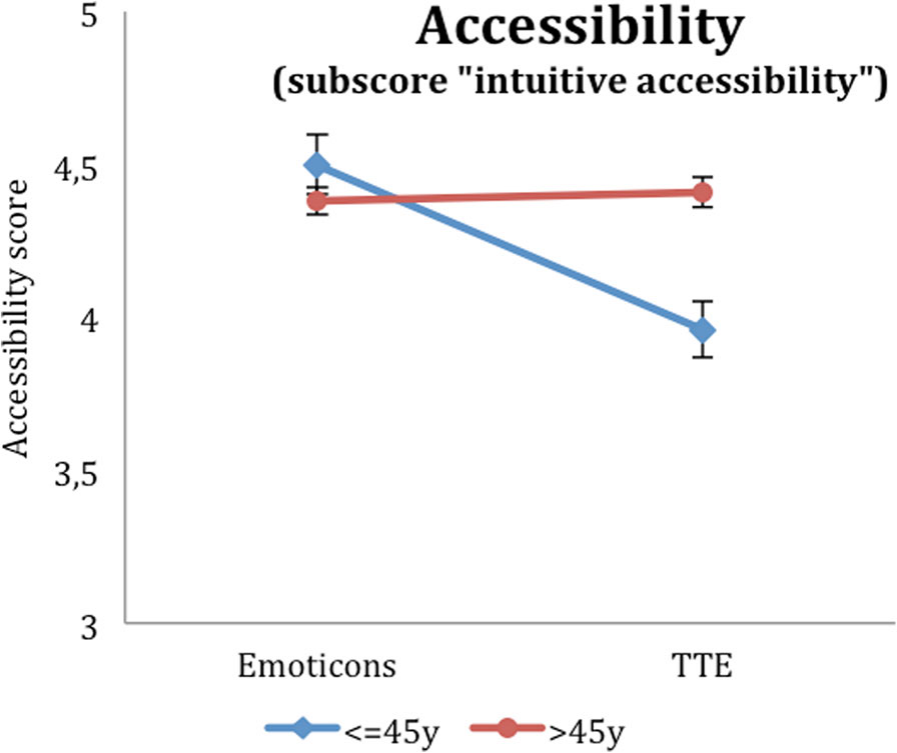
Accessibility as a function of age-group and illustration

**Fig. 6:**
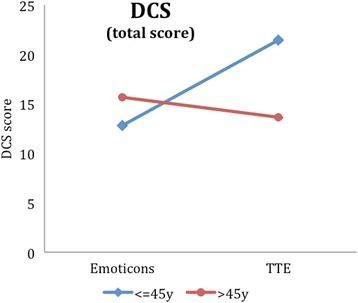
DCS (total score) as a function of age-group and illustration

**Fig. 7:**
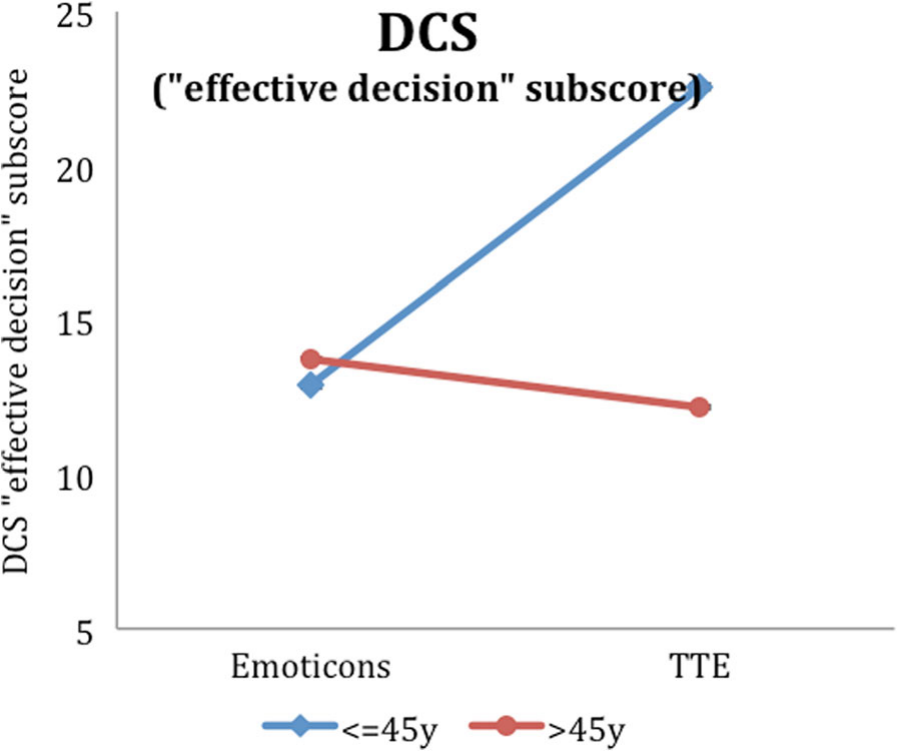
DCS (effective decision subscore) as a function of age-group and illustration

**Fig. 8:**
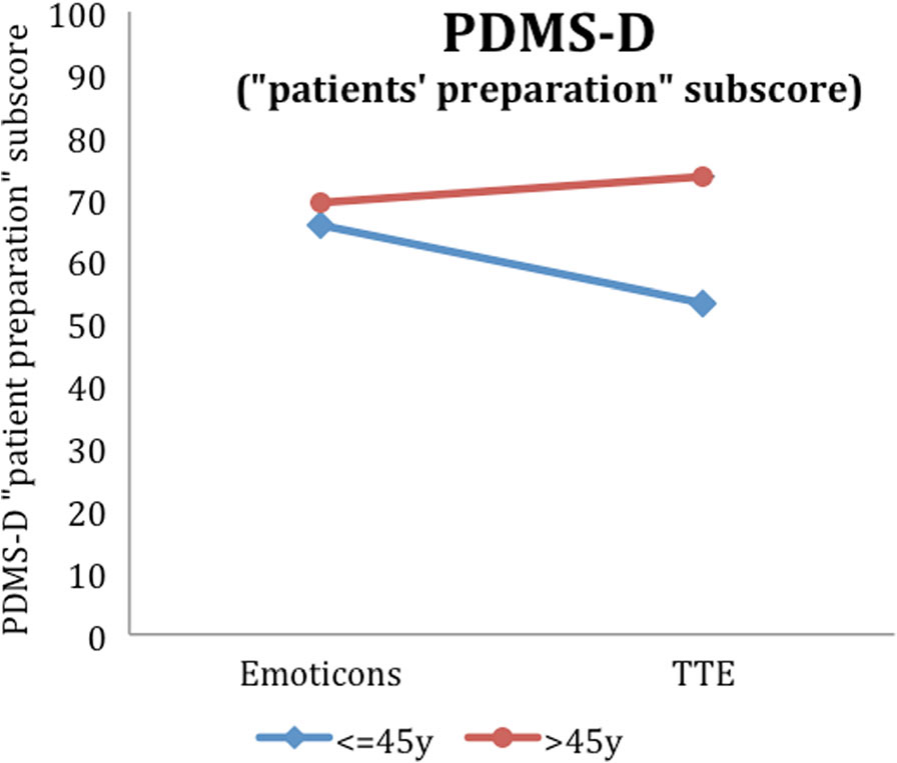
PDMS-D (subscore patients’s preparation for the GP consultation) as a function of age-group and illustration

In the explorative analyses, interactions of age and illustration were tested in a two-factorial ANOVA. Here, we could find four other outcome variables with a significant interaction, i.e. the intuitive accessibility (Additional file 2: Table S2, Fig. 5), the total DCS score (Additional file 3: Table S3, Fig. 6), the DCS effective decision subscore (Additional file 4: Table S4, Fig. 7) and the PDMS-D subscore “preparation for the GP consultation” (Additional file 5: Table S5, Fig. 8). Here, the superiority of the TTE could be confirmed in both patient groups with respect to age, in which the difference in the group of younger patients is higher. For instance, looking at the DCS, scores in the group of younger patients are remarkably higher than scores for older patients for the TTE, while they differ less for the emoticons (Additional file 3: Table S3). This accounts for the total DCS as well as for all DCS subscales and represents a higher level of decisional conflict.

## Discussion

### Summary

Regarding our primary outcome PEF-FB9 the new TTE illustration is not inferior compared to the emoticons (whole sample). Furthermore, the non-inferiority of the innovative TTE could be confirmed for all secondary outcome variables. The explorative comparison of young and old patients even indicates advantages, e.g. a higher risk perception, of the TTE in younger patient groups (below 46 years of age).

### Strengths and limitations

Our study has some limitations: First, the reasons for higher scores in the DCS and the PDMS-D scale in the younger population are not fully explained. We can only speculate about these results and reasons for the differences. Further qualitative research especially looking at younger patients and their motivation would be necessary to shed light on these issues. This is directly related to and caused by the second limitation. Our study sample was drawn from practice attenders with a need to discuss cardiovascular prevention. It therefore reflects the demographic and morbidity features of this group. As a result, younger persons were underrepresented. In order to replicate and strengthen these results as well as further investigate the younger population and the mechanisms that underlie the results, which were mentioned and discussed above, future studies should include higher numbers of young patients. Third, the TTE representation in our arriba^TM^ study version did not allow for all combinations of risk-reducing interventions that are possible. As mentioned above, a complex Markov model is underlying this risk format, but could illustrate only one intervention in isolation but no combination of several treatments. Those combinations are not very likely, but nevertheless possible and the decrease of risk needs to be illustrated for the patient.

Despite these limitations we still think that our study provides valid estimates regarding the outcome measure. Our study is the first study to investigate real patients in the setting of a GP consultation and regarding CV decisions concerning themselves looking at the PEF-FB 9 and the other secondary outcomes mentioned and discussed in detail above. In fact, issues regarding their own cardiovascular risks and preventive options were discussed and real decisions were taken.

### Comparison with existing literature

Literature on the effect of lifetime risk predictions with respect to cardiovascular risk on patients’ decision-making is scarce. Hence, it is challenging to assess how risk formats impact on the patient’s motivation to participate in the decision-making process. Several studies have investigated the impact of a TTE illustration. Advantages with respect to understanding compared to other formats could be shown [40–43]. Compared to our work, previous studies did not consider other representations. A Danish study asked participants for the level of understanding and acceptance of a fictional drug treatment in order to postpone heart attacks. The information was delivered in a verbal time-to-event exercise with varying timeframes of postponement. There was no comparison with another risk format but overall level of understanding was high with about 81% of all participants judging the information as “not difficult” [43]. Furthermore, studies in this filed of research were performed by a web-based survey and not in the setting of a GP consultation.

Carling et al. presented the benefit of a fictional antibiotic drug in a web-based survey. In detail, one of several formats was shown to healthy individuals, i.e. the percentage of persons with improved symptoms after three days. The presentation was either done as icon arrays with smileys, horizontal bars or duration of symptoms as a horizontal bar (i.e. time until sore throat has subsided). The latter display was judged the most understandable.

### Implications for research and practice

Focusing on younger patients, especially patients at risk, little research has been performed with respect to cardiovascular risk prevention in general practice. It seems quite obvious that counselling young patients with a ten-year-absolute-risk information might lead to an underestimation of their lifetime risk, i.e. a significant lifetime risk will not be detected, even in high-risk patients. Thus, behavioural change is unlikely to happen in these cases and prevention mechanisms cannot work properly. For instance, looking at those younger patients we could first demonstrate, that the TTE - as mentioned above – leads to a higher risk perception immediately after the GP consultation. We assume that this higher risk perception is due to the fact that a risk, which has not been considered previously, becomes obvious only in the context of the GP consultation with TTE. For younger people, this is seems to be much more relevant than the conventional ten-year prognosis.

Next, we could show that the decisional conflict was considerably higher in younger patients confronted with the TTE compared to the emoticons. In our view, this shows that the TTE representation in arriba^TM^ raises an issue that the patient has not been aware of prior the consultation. In this case, raised decisional conflict should not necessarily be seen as negative. Future studies, especially qualitative investigations and quantitative surveys with prolonged follow-up are needed to explore these findings further.

Furthermore, results indicate lower scores in the PDMS-D in the TTE group, i.e. the experienced degree of information is lower in young patients counselled with the TTE. We can speculate about the reasons for this finding. Maybe young patients did not understand the representation that well, or not enough options for action could be demonstrated. Hence, looking at the two dimensions of the PDMS-D, the finding becomes clearer. The difference is primarily caused by significantly lower scores in the “preparation for GP consultation” sub-scores and is therefore not first-order related to the representation itself.

## Conclusion

The results of our study show that the illustration of the event-free survival (TTE) is appropriate for cardiovascular risk information. Moreover, this illustration offers the possibility to counsel younger patients adequately, so that relevant preventive actions can be started despite absolute risks still being small. The latter is not sufficiently the case with the risk representations that are currently available and all provide a 10-year prognosis. Whether the higher decisional conflict in the TTE group is generated by the improved risk perception, has to be proven in future studies.

## Additional files

Additional file 1: Table S1. Risk perception of patients. Additional file 1: Table S1 shows the risk perception depending on risk representation and age-group. (DOCX 15 kb)
Additional file 2: Table S2. Accessibility, intuitive accessibility subscore. Additional file 2: Table S2 shows the “intuitive accessibility” subscore of the accessibility assessment depending on risk representation and age-group. (DOCX 14 kb)
Additional file 3: Table S3. Decisional Conflict Scale (DCS), total score. Additional file 3: Table S3 shows the total score of the decisional conflict scale (DCS) depending on risk representation and age-group. (DOCX 15 kb)
Additional file 4: Table S4. Decisional Conflict Scale (DCS), effective decision subscore. Additional file 4: Table S4 shows the effective decision subscore of the decisional conflict scale (DCS) depending on risk representation and age-group. (DOCX 15 kb)
Additional file 5: Table S5. DCS, effective decision subscore. Additional file 5: Table S5 shows the patients’ preparation for the GP consultation subscore of the Preparation for Decision-Making Scale (PDMS-D). (DOCX 15 kb)

## Abbreviations

DCS: Decisional Conflict Scale
DMP: Disease Management Program
GP: General practitioner
OptRisk: Optimizing-Risk-Communication
PDMS: Preparation for Decision Making Scale
PDMS-D: German version of the Preparation for Decision Making Scale
PEF-FB-9: Partizipative Entscheidungsfindung-Fragebogen-9 (German questionnaire on the participation in the shared decision-making process
SDM: Shared decision-making
TTE: Time-to-event
