# Supplementary material for: Time-to-event versus ten-year-absolute-risk in cardiovascular risk prevention – does it make a difference? Results from the Optimizing-Risk-Communication (OptRisk) randomized-controlled trial
Source: BMC Med Inform Decis Mak. 2016 Nov 29;16:152. doi: 10.1186/s12911-016-0393-1 (PMC5129612; doi:10.1186/s12911-016-0393-1)
Supplement: Additional file 1: Table S1. — Risk perception of patients. Additional file 1: Table S1 shows the risk perception depending on risk representation and age-group. (DOCX 15 kb) [file 12911_2016_393_MOESM1_ESM.docx]

**Additional file 1: Table S1.** Risk perception of patients

|  | age | illustration | n | Mean (sd) | p-value t-test  main effect | p-value interaction |
| --- | --- | --- | --- | --- | --- | --- |
| **Risk perception** | <=45 y | Emoticons | 16 | 2,38 (2.5) | .001 | 0.008 |
|  |  | TTE | 23 | 5,65 (3.0) |  |  |
|  | >45 y | Emoticons | 130 | 4,27 (2.25) | .006 |  |
|  |  | TTE | 134 | 5,14 (2.82) |  |  |
