# Supplementary material for: Time-to-event versus ten-year-absolute-risk in cardiovascular risk prevention – does it make a difference? Results from the Optimizing-Risk-Communication (OptRisk) randomized-controlled trial
Source: BMC Med Inform Decis Mak. 2016 Nov 29;16:152. doi: 10.1186/s12911-016-0393-1 (PMC5129612; doi:10.1186/s12911-016-0393-1)
Supplement: Additional file 2: Table S2. — Accessibility, intuitive accessibility subscore. Additional file 2: Table S2 shows the “intuitive accessibility” subscore of the accessibility assessment depending on risk representation and age-group. (DOCX 14 kb) [file 12911_2016_393_MOESM2_ESM.docx]

**Additional file 2: Table S2.** Accessibility

|  | age | illustration | n | Mean (sd) | p-value t-test  main effect | p-value interaction |
| --- | --- | --- | --- | --- | --- | --- |
| **Accessibility** Subscore  „intuitive accessibility“ | <=45 y | Emoticons | 16 | 4,50 (.632) | .041 | 0.028 |
|  |  | TTE | 23 | 3,96 (.878) |  |  |
|  | >45 y | Emoticons | 130 | 4,38 (.719) | .775 |  |
|  |  | TTE | 134 | 4,41 (.748) |  |  |
