# Supplementary material for: Time-to-event versus ten-year-absolute-risk in cardiovascular risk prevention – does it make a difference? Results from the Optimizing-Risk-Communication (OptRisk) randomized-controlled trial
Source: BMC Med Inform Decis Mak. 2016 Nov 29;16:152. doi: 10.1186/s12911-016-0393-1 (PMC5129612; doi:10.1186/s12911-016-0393-1)
Supplement: Additional file 3: Table S3. — Decisional Conflict Scale (DCS), total score. Additional file 3: Table S3 shows the total score of the decisional conflict scale (DCS) depending on risk representation and age-group. (DOCX 15 kb) [file 12911_2016_393_MOESM3_ESM.docx]

**Additional file 3: Table S3.** Decisional Conflict Scale (DCS), total score

|  | age | illustration | n | Mean (sd) | p-value t-test  main effect | p-value interaction |
| --- | --- | --- | --- | --- | --- | --- |
| **DCS**  (Total Score) | <=45 y | Emoticons | 16 | 12,81 (15.22) | .117 | 0.024 |
|  |  | TTE | 23 | 21,43 (16.25) |  |  |
|  | >45 y | Emoticons | 130 | 15,7 (13.83) | .201 |  |
|  |  | TTE | 134 | 13,63 (11.25) |  |  |
