# Supplementary material for: Time-to-event versus ten-year-absolute-risk in cardiovascular risk prevention – does it make a difference? Results from the Optimizing-Risk-Communication (OptRisk) randomized-controlled trial
Source: BMC Med Inform Decis Mak. 2016 Nov 29;16:152. doi: 10.1186/s12911-016-0393-1 (PMC5129612; doi:10.1186/s12911-016-0393-1)
Supplement: Additional file 4: Table S4. — Decisional Conflict Scale (DCS), effective decision subscore. Additional file 4: Table S4 shows the effective decision subscore of the decisional conflict scale (DCS) depending on risk representation and age-group. (DOCX 15 kb) [file 12911_2016_393_MOESM4_ESM.docx]

**Additional file 4: Table S4.** DCS, effective decision subscore

|  | age | illustration | n | Mean (sd) | p-value t-test  main effect | p-value interaction |
| --- | --- | --- | --- | --- | --- | --- |
| **DCS** Effective Decision Subscore | <=45 y | Emoticons | 16 | 12,89 (16.05) | .107 | 0.037 |
|  |  | TTE | 23 | 22,55 (19.19) |  |  |
|  | >45 y | Emoticons | 130 | 13,76 (15.88) | .392 |  |
|  |  | TTE | 134 | 12,16 (14.02) |  |  |
