# Supplementary material for: Time-to-event versus ten-year-absolute-risk in cardiovascular risk prevention – does it make a difference? Results from the Optimizing-Risk-Communication (OptRisk) randomized-controlled trial
Source: BMC Med Inform Decis Mak. 2016 Nov 29;16:152. doi: 10.1186/s12911-016-0393-1 (PMC5129612; doi:10.1186/s12911-016-0393-1)
Supplement: Additional file 5: Table S5. — DCS, effective decision subscore. Additional file 5: Table S5 shows the patients’ preparation for the GP consultation subscore of the Preparation for Decision-Making Scale (PDMS-D). (DOCX 15 kb) [file 12911_2016_393_MOESM5_ESM.docx]

**Additional file 5: Table S5.** Preparation for Decision-Making Scale (PDMS-D), subscore patients’ preparation for the GP consultation

|  | age | illustration | n | Mean (sd) | p-value ttest  main effect | p-value interaction |
| --- | --- | --- | --- | --- | --- | --- |
| **PDMS-D**  _Subscore  “Preparation for GP consultation” (0-100) | <=45 y | Emoticons | 16 | 65,63 (27.36) | .163 | 0.048 |
|  |  | TTE | 23 | 53,26 (26.2) |  |  |
|  | >45 y | Emoticons | 130 | 69,33 (23.63) | .150 |  |
|  |  | TTE | 134 | 73,62 (23.56) |  |  |
